# Trichlorido(5,5′-dimethyl-2,2′-bipyridine-κ^2^ *N*,*N*′)(dimethyl­formamide-κ*O*)indium(III) hemihydrate

**DOI:** 10.1107/S1600536812044698

**Published:** 2012-11-03

**Authors:** Sadif A. Shirvan, Sara Haydari Dezfuli, Fereydoon Khazali, Manouchehr Aghajeri, Ali Borsalani

**Affiliations:** a Department of Chemistry, Omidieh Branch, Islamic Azad University, Omidieh, Iran; b Department of Petroleum Engineering, Omidieh Branch, Islamic Azad University, Omidieh, Iran

## Abstract

The asymmetric unit of the title compound, [InCl_3_(C_12_H_12_N_2_)(C_3_H_7_NO)]·0.5H_2_O, contains two independent In^III^ complex mol­ecules with similar structures and one lattice water mol­ecule. In each complex mol­ecule, the In^III^ atom is six-coordinated in a distorted octa­hedral geometry, formed by two N atoms from the chelating 5,5′-dimethyl-2,2′-bipyridine ligand, one O atom from a dimethyl­formamide and three *facial* Cl atoms. In the crystal, the lattice water mol­ecule is linked to the complex mol­ecules *via* O—H⋯Cl hydrogen bonds. Further weak C—H⋯Cl and C—H⋯O hydrogen bonds result in the formation of a three-dimensional structure.

## Related literature

For related structures, see: Albada *et al.* (2004 ▶); Alizadeh *et al.* (2010 ▶); Amani *et al.* (2007 ▶, 2009 ▶); Kalateh *et al.* (2008 ▶); Khalighi *et al.* (2008 ▶); Shirvan & Haydari Dezfuli (2012 ▶); Tadayon Pour *et al.* (2008 ▶).
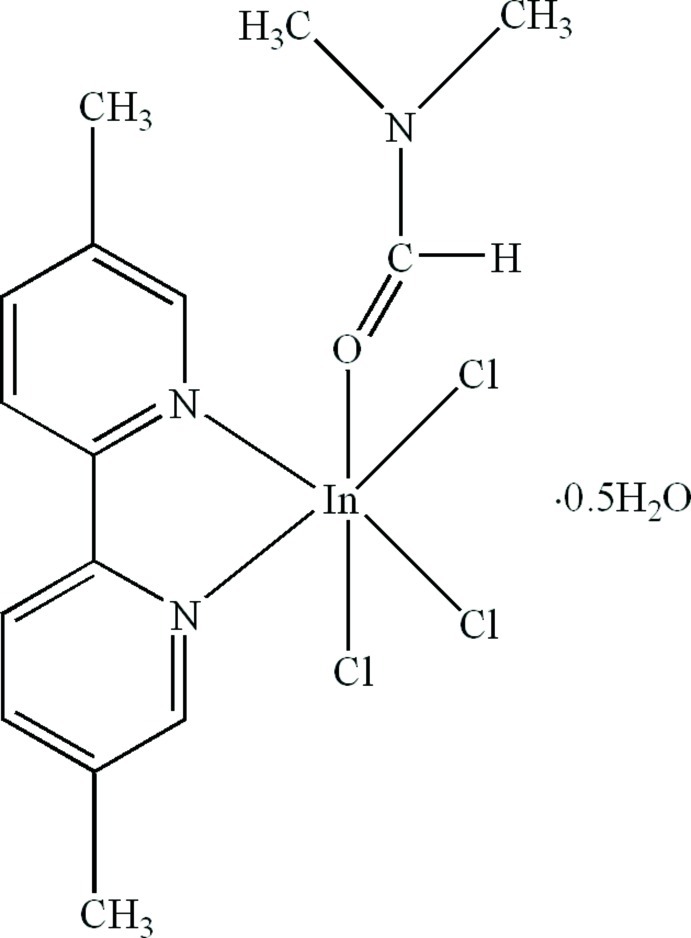


## Experimental

### 

#### Crystal data

[InCl_3_(C_12_H_12_N_2_)(C_3_H_7_NO)]·0.5H_2_O
*M* *_r_* = 487.51
Triclinic, 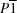

*a* = 11.3021 (5) Å
*b* = 11.4445 (5) Å
*c* = 15.0860 (6) Å
α = 91.089 (4)°
β = 96.024 (3)°
γ = 96.448 (4)°
*V* = 1927.34 (14) Å^3^
*Z* = 4
Mo *K*α radiation
μ = 1.65 mm^−1^
*T* = 173 K
0.32 × 0.30 × 0.25 mm

#### Data collection

Bruker APEXII CCD area-detector diffractometer
Absorption correction: multi-scan (*SADABS*; Bruker, 2001 ▶) *T*_min_ = 0.601, *T*_max_ = 0.688
15402 measured reflections
7524 independent reflections
6051 reflections with *I* > 2σ(*I*)
*R*_int_ = 0.036

#### Refinement

*R*[*F* ^2^ > 2σ(*F* ^2^)] = 0.034
*wR*(*F* ^2^) = 0.067
*S* = 1.03
7524 reflections
437 parameters
3 restraints
H atoms treated by a mixture of independent and constrained refinement
Δρ_max_ = 0.57 e Å^−3^
Δρ_min_ = −0.54 e Å^−3^

### 

Data collection: *APEX2* (Bruker, 2007 ▶); cell refinement: *SAINT* (Bruker, 2007 ▶); data reduction: *SAINT*; program(s) used to solve structure: *SHELXTL* (Sheldrick, 2008 ▶); program(s) used to refine structure: *SHELXTL*; molecular graphics: *SHELXTL*; software used to prepare material for publication: *SHELXTL*.

## Supplementary Material

Crystal structure: contains datablock(s) I, global. DOI: 10.1107/S1600536812044698/xu5639sup1.cif

Structure factors: contains datablock(s) I. DOI: 10.1107/S1600536812044698/xu5639Isup2.hkl

Additional supplementary materials:  crystallographic information; 3D view; checkCIF report

## Figures and Tables

**Table 1:** Selected bond lengths (Å)

In1—Cl1	2.4185 (9)
In1—Cl2	2.4227 (9)
In1—Cl3	2.4496 (9)
In1—O1	2.267 (2)
In1—N1	2.287 (3)
In1—N2	2.301 (3)
In2—Cl4	2.4737 (9)
In2—Cl5	2.4327 (10)
In2—Cl6	2.4326 (9)
In2—O2	2.202 (3)
In2—N4	2.292 (3)
In2—N5	2.316 (3)

**Table 2:** Hydrogen-bond geometry (Å, °)

*D*—H⋯*A*	*D*—H	H⋯*A*	*D*⋯*A*	*D*—H⋯*A*
O3—H3*D*⋯Cl5^i^	0.84 (3)	2.49 (3)	3.326 (5)	174 (3)
O3—H3*E*⋯Cl3^ii^	0.84 (2)	2.40 (2)	3.183 (5)	156 (3)
C5—H5⋯Cl4	0.93	2.69	3.620 (3)	175
C8—H8⋯Cl4	0.93	2.69	3.574 (4)	160
C9—H9⋯Cl4^iii^	0.93	2.78	3.657 (3)	158
C20—H20⋯O3^iv^	0.93	2.60	3.526 (6)	178
C23—H23⋯O3^iv^	0.93	2.49	3.416 (6)	173

Symmetry codes: (i) 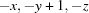; (ii) 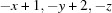; (iii) 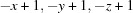; (iv) 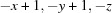.

**Fig. 1.**
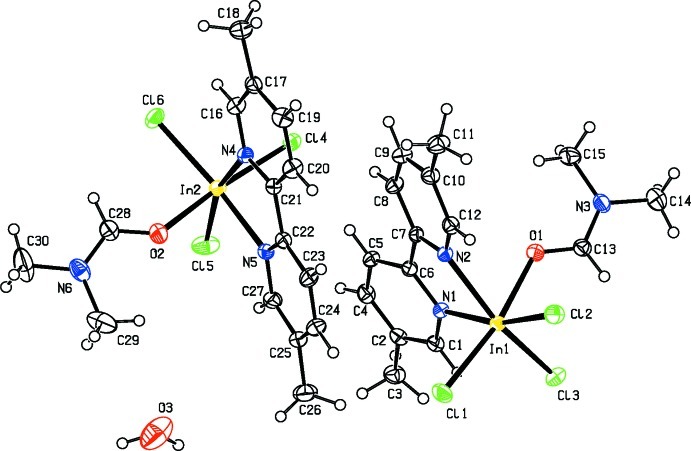
The molecular structure of the title molecule, with the atom-numbering scheme. Displacement ellipsoids are drawn at the 50% probability level.

**Fig. 2.**
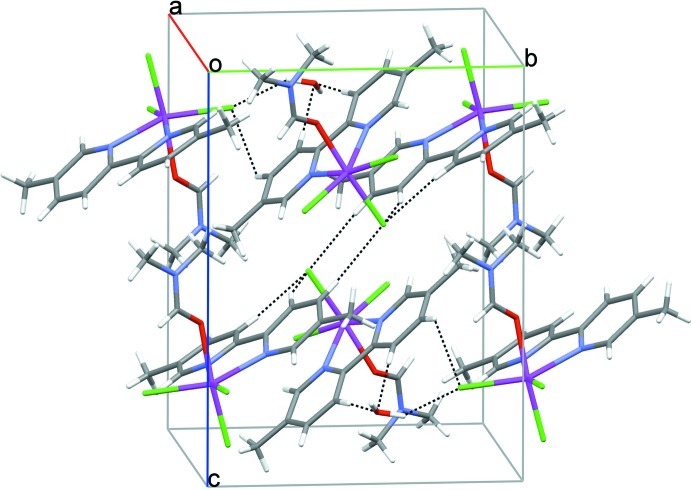
Unit-cell packing diagram for title molecule. Hydrogen bonds are shown as dashed lines.

## supplementary crystallographic information

### Comment

Recently, we reported the synthes and crystal structure of [Cd(5,5'-dmbpy)(µ-Br)_2_]_n_, (Shirvan & Haydari Dezfuli, 2012) [where 5,5'-dmbpy is 5,5'-dimethyl-2,2'-bipyridine]. 5,5'-Dimethyl-2,2'-bipyridine is a good bidentate ligand, and numerous complexes with 5,5'-dmbipy have been prepared, such as that of zinc (Khalighi *et al.*, 2008), indium (Kalateh *et al.*, 2008), iron (Amani *et al.*, 2007), platin (Amani *et al.*, 2009), copper (Albada *et al.*, 2004) and mercury (Tadayon Pour *et al.*, 2008; Alizadeh *et al.*, 2010). Here, we report the synthesis and structure of the title compound.

The asymmetric unit of the title compound, (Fig. 1), contains two crystallographically independent [In(C_12_H_12_N_2_)Cl_3_(C_3_H_7_NO)] molecules and one water solvent molecule. The In^III^ atom is six-coordinated in a distorted octahedral configuration by two N atoms from the chelating 5,5'-dimethyl-2,2'-bipyridine ligand, one O atom from a dimethylformamide and three Cl atoms. The In—Cl, In—O and In—N bond lengths and angles are collected in Table 1.

In the crystal structure, intermolecular O—H···Cl and C—H···O and C—H···Cl hydrogen bonds link the molecules (Fig. 2 & Table 2).

### Experimental

For the preparation of the title compound, a solution of 5,5'-dimethyl-2,2'-bipyridine (0.30 g, 1.65 mmol) in methanol (10 ml) was added to a solution of InCl_3_.4H_2_O (0.48 g, 1.65 mmol) in methanol (10 ml) and the resulting colorless solution was stirred for 20 min at 323 K. The suitable crystals for X-ray diffraction experiment were obtained by methanol diffusion to a colorless solution in dimethylformamide. Suitable crystals were isolated after one week (yield; 0.58 g, 72.1%).

### Refinement

Water H atoms were located in a difference Fourier map and refined with O—H distance of 0.84 (2) Å, U_iso_(H) = 0.1 Å^2^. Other H atoms were positioned geometrically with C—H = 0.93 Å for aromatics H and 0.96 Å for methyl H atoms constrained to ride on their parent atoms, *U*_iso_(H) = 1.2*U*_eq_(C).

### Crystal data

**Table:**

[InCl_3_(C_12_H_12_N_2_)(C_3_H_7_NO)]·0.5H_2_O	*Z* = 4
*M**_r_* = 487.51	*F*(000) = 972
Triclinic, *P*1	*D*_x_ = 1.680 Mg m^−^^3^
Hall symbol: -P 1	Mo *K*α radiation, λ = 0.71073 Å
*a* = 11.3021 (5) Å	Cell parameters from 15402 reflections
*b* = 11.4445 (5) Å	θ = 2.4–26.0°
*c* = 15.0860 (6) Å	µ = 1.65 mm^−^^1^
α = 91.089 (4)°	*T* = 173 K
β = 96.024 (3)°	Block, colorless
γ = 96.448 (4)°	0.32 × 0.30 × 0.25 mm
*V* = 1927.34 (14) Å^3^	

### Data collection

**Table:**

Bruker APEXII CCD area-detector diffractometer	7524 independent reflections
Radiation source: fine-focus sealed tube	6051 reflections with *I* > 2σ(*I*)
Graphite monochromator	*R*_int_ = 0.036
ω scans	θ_max_ = 26.0°, θ_min_ = 2.4°
Absorption correction: multi-scan (*SADABS*; Bruker, 2001)	*h* = −13→13
*T*_min_ = 0.601, *T*_max_ = 0.688	*k* = −14→14
15402 measured reflections	*l* = −18→18

### Refinement

**Table:**

Refinement on *F*^2^	Primary atom site location: structure-invariant direct methods
Least-squares matrix: full	Secondary atom site location: difference Fourier map
*R*[*F*^2^ > 2σ(*F*^2^)] = 0.034	Hydrogen site location: inferred from neighbouring sites
*wR*(*F*^2^) = 0.067	H atoms treated by a mixture of independent and constrained refinement
*S* = 1.03	*w* = 1/[σ^2^(*F*_o_^2^) + (0.0309*P*)^2^ + 0.5815*P*] where *P* = (*F*_o_^2^ + 2*F*_c_^2^)/3
7524 reflections	(Δ/σ)_max_ = 0.001
437 parameters	Δρ_max_ = 0.57 e Å^−^^3^
3 restraints	Δρ_min_ = −0.54 e Å^−^^3^

### Special details

**Table:**

Geometry. All e.s.d.'s (except the e.s.d. in the dihedral angle between two l.s. planes) are estimated using the full covariance matrix. The cell e.s.d.'s are taken into account individually in the estimation of e.s.d.'s in distances, angles and torsion angles; correlations between e.s.d.'s in cell parameters are only used when they are defined by crystal symmetry. An approximate (isotropic) treatment of cell e.s.d.'s is used for estimating e.s.d.'s involving l.s. planes.
Refinement. Refinement of *F*^2^ against ALL reflections. The weighted *R*-factor *wR* and goodness of fit *S* are based on *F*^2^, conventional *R*-factors *R* are based on *F*, with *F* set to zero for negative *F*^2^. The threshold expression of *F*^2^ > σ(*F*^2^) is used only for calculating *R*-factors(gt) *etc*. and is not relevant to the choice of reflections for refinement. *R*-factors based on *F*^2^ are statistically about twice as large as those based on *F*, and *R*- factors based on ALL data will be even larger.

### Fractional atomic coordinates and isotropic or equivalent isotropic displacement parameters (Å^2^)

**Table:**

	*x*	*y*	*z*	*U*_iso_*/*U*_eq_	
C1	0.5123 (3)	1.0246 (3)	0.2042 (2)	0.0251 (7)	
H1	0.5450	1.0890	0.1746	0.030*	
C2	0.3899 (3)	1.0132 (3)	0.2121 (2)	0.0255 (7)	
C3	0.3115 (3)	1.1025 (4)	0.1753 (3)	0.0341 (9)	
H3C	0.2652	1.1270	0.2206	0.041*	
H3B	0.2587	1.0682	0.1253	0.041*	
H3A	0.3606	1.1694	0.1564	0.041*	
C4	0.3441 (3)	0.9152 (3)	0.2551 (2)	0.0277 (8)	
H4	0.2625	0.9031	0.2608	0.033*	
C5	0.4183 (3)	0.8350 (3)	0.2899 (2)	0.0249 (7)	
H5	0.3875	0.7697	0.3194	0.030*	
C6	0.5403 (3)	0.8539 (3)	0.2797 (2)	0.0222 (7)	
C7	0.6250 (3)	0.7694 (3)	0.3113 (2)	0.0206 (7)	
C8	0.5969 (3)	0.6830 (3)	0.3715 (2)	0.0261 (7)	
H8	0.5227	0.6760	0.3935	0.031*	
C9	0.6800 (3)	0.6076 (3)	0.3984 (2)	0.0293 (8)	
H9	0.6626	0.5510	0.4400	0.035*	
C10	0.7899 (3)	0.6162 (3)	0.3633 (2)	0.0272 (8)	
C11	0.8797 (4)	0.5334 (4)	0.3906 (3)	0.0377 (9)	
H11C	0.8845	0.5243	0.4539	0.045*	
H11B	0.9567	0.5645	0.3744	0.045*	
H11A	0.8554	0.4583	0.3609	0.045*	
C12	0.8112 (3)	0.7047 (3)	0.3039 (2)	0.0252 (7)	
H12	0.8839	0.7117	0.2797	0.030*	
C13	0.8628 (3)	1.0800 (3)	0.4086 (2)	0.0269 (8)	
H13	0.9023	1.1386	0.3770	0.032*	
C14	0.9446 (4)	1.1909 (4)	0.5437 (3)	0.0495 (12)	
H14C	1.0074	1.1657	0.5842	0.059*	
H14B	0.8924	1.2313	0.5766	0.059*	
H14A	0.9788	1.2430	0.5017	0.059*	
C15	0.8199 (4)	0.9974 (4)	0.5493 (3)	0.0422 (10)	
H15A	0.7973	0.9266	0.5134	0.051*	
H15B	0.7501	1.0237	0.5706	0.051*	
H15C	0.8756	0.9819	0.5990	0.051*	
C16	0.4078 (3)	0.2947 (3)	0.3691 (2)	0.0267 (7)	
H16	0.3432	0.2736	0.4012	0.032*	
C17	0.5101 (3)	0.2392 (3)	0.3877 (2)	0.0272 (8)	
C18	0.5177 (4)	0.1442 (3)	0.4548 (3)	0.0359 (9)	
H18A	0.4572	0.0799	0.4372	0.043*	
H18B	0.5054	0.1751	0.5123	0.043*	
H18C	0.5953	0.1170	0.4577	0.043*	
C19	0.6052 (3)	0.2748 (3)	0.3395 (2)	0.0314 (8)	
H19	0.6763	0.2410	0.3498	0.038*	
C20	0.5954 (3)	0.3597 (3)	0.2764 (2)	0.0312 (8)	
H20	0.6594	0.3832	0.2442	0.037*	
C21	0.4897 (3)	0.4096 (3)	0.2615 (2)	0.0232 (7)	
C22	0.4726 (3)	0.5031 (3)	0.1956 (2)	0.0236 (7)	
C23	0.5582 (3)	0.5398 (3)	0.1397 (2)	0.0317 (8)	
H23	0.6299	0.5067	0.1427	0.038*	
C24	0.5356 (4)	0.6265 (4)	0.0791 (2)	0.0341 (9)	
H24	0.5925	0.6517	0.0411	0.041*	
C25	0.4292 (3)	0.6756 (3)	0.0749 (2)	0.0301 (8)	
C26	0.4010 (4)	0.7694 (4)	0.0104 (3)	0.0460 (11)	
H26C	0.4709	0.8250	0.0084	0.055*	
H26B	0.3371	0.8091	0.0293	0.055*	
H26A	0.3771	0.7341	−0.0479	0.055*	
C27	0.3478 (3)	0.6325 (3)	0.1325 (2)	0.0287 (8)	
H27	0.2744	0.6625	0.1295	0.034*	
C28	0.1038 (3)	0.2848 (4)	0.1282 (2)	0.0322 (8)	
H28	0.0720	0.2356	0.1703	0.039*	
C29	0.1028 (5)	0.3466 (5)	−0.0221 (3)	0.0639 (15)	
H29C	0.1406	0.3045	−0.0648	0.077*	
H29B	0.1591	0.4090	0.0058	0.077*	
H29A	0.0352	0.3791	−0.0518	0.077*	
C30	−0.0240 (5)	0.1663 (6)	0.0160 (3)	0.0706 (18)	
H30A	−0.0455	0.1230	0.0669	0.085*	
H30B	0.0105	0.1162	−0.0235	0.085*	
H30C	−0.0941	0.1933	−0.0146	0.085*	
N1	0.5854 (2)	0.9478 (2)	0.23712 (18)	0.0219 (6)	
N2	0.7329 (2)	0.7812 (2)	0.27921 (17)	0.0209 (6)	
N3	0.8759 (3)	1.0879 (3)	0.49582 (19)	0.0290 (7)	
N4	0.3964 (2)	0.3764 (2)	0.30794 (18)	0.0229 (6)	
N5	0.3691 (3)	0.5500 (3)	0.19222 (18)	0.0234 (6)	
N6	0.0629 (3)	0.2669 (3)	0.0452 (2)	0.0378 (8)	
O1	0.7993 (2)	0.9967 (2)	0.36637 (15)	0.0309 (6)	
O2	0.1854 (2)	0.3663 (2)	0.15434 (17)	0.0375 (6)	
O3	0.1661 (4)	0.5577 (4)	−0.1491 (4)	0.0903 (15)	
Cl1	0.72933 (9)	0.90119 (10)	0.06210 (6)	0.0395 (2)	
Cl2	0.99623 (8)	0.93947 (9)	0.22712 (6)	0.0333 (2)	
Cl3	0.79761 (8)	1.17277 (8)	0.19951 (6)	0.0313 (2)	
Cl4	0.29650 (8)	0.59312 (8)	0.41650 (6)	0.03024 (19)	
Cl5	0.08798 (9)	0.60500 (10)	0.22539 (7)	0.0435 (3)	
Cl6	0.10673 (8)	0.31911 (9)	0.35452 (6)	0.0355 (2)	
In1	0.78415 (2)	0.95952 (2)	0.217222 (15)	0.02104 (7)	
In2	0.22743 (2)	0.46926 (2)	0.280738 (15)	0.02290 (7)	
H3D	0.099 (2)	0.521 (3)	−0.168 (3)	0.100*	
H3E	0.155 (3)	0.6286 (12)	−0.156 (3)	0.100*	

### Atomic displacement parameters (Å^2^)

**Table:**

	*U*^11^	*U*^22^	*U*^33^	*U*^12^	*U*^13^	*U*^23^
C1	0.0259 (18)	0.0258 (18)	0.0228 (16)	0.0032 (14)	−0.0012 (14)	0.0023 (14)
C2	0.0253 (18)	0.0315 (19)	0.0194 (16)	0.0047 (15)	−0.0001 (13)	−0.0012 (14)
C3	0.029 (2)	0.040 (2)	0.034 (2)	0.0100 (17)	0.0012 (16)	0.0072 (17)
C4	0.0188 (17)	0.037 (2)	0.0276 (17)	0.0032 (15)	0.0036 (14)	−0.0021 (15)
C5	0.0258 (18)	0.0259 (18)	0.0227 (16)	−0.0021 (14)	0.0062 (14)	0.0030 (14)
C6	0.0249 (18)	0.0227 (17)	0.0184 (15)	−0.0008 (14)	0.0042 (13)	−0.0016 (13)
C7	0.0214 (17)	0.0207 (17)	0.0189 (15)	−0.0019 (13)	0.0034 (13)	−0.0014 (13)
C8	0.0228 (18)	0.0287 (19)	0.0278 (17)	0.0010 (14)	0.0087 (14)	0.0035 (14)
C9	0.034 (2)	0.0283 (19)	0.0262 (18)	0.0044 (16)	0.0052 (15)	0.0079 (15)
C10	0.0265 (19)	0.0279 (19)	0.0275 (17)	0.0052 (15)	0.0022 (14)	0.0025 (14)
C11	0.034 (2)	0.039 (2)	0.043 (2)	0.0098 (18)	0.0055 (18)	0.0156 (18)
C12	0.0220 (18)	0.0290 (19)	0.0244 (17)	0.0020 (14)	0.0032 (13)	−0.0009 (14)
C13	0.0236 (18)	0.033 (2)	0.0238 (17)	0.0024 (15)	0.0026 (14)	0.0021 (15)
C14	0.059 (3)	0.049 (3)	0.034 (2)	−0.010 (2)	−0.003 (2)	−0.006 (2)
C15	0.043 (2)	0.059 (3)	0.0232 (18)	−0.006 (2)	0.0073 (17)	0.0034 (18)
C16	0.0285 (19)	0.0246 (18)	0.0280 (18)	0.0033 (14)	0.0071 (14)	0.0036 (14)
C17	0.031 (2)	0.0233 (18)	0.0261 (17)	0.0031 (15)	−0.0029 (15)	−0.0003 (14)
C18	0.040 (2)	0.032 (2)	0.037 (2)	0.0107 (17)	0.0023 (17)	0.0090 (17)
C19	0.031 (2)	0.030 (2)	0.035 (2)	0.0141 (16)	0.0039 (16)	0.0006 (16)
C20	0.0260 (19)	0.033 (2)	0.036 (2)	0.0077 (16)	0.0073 (16)	0.0028 (16)
C21	0.0237 (18)	0.0223 (17)	0.0227 (16)	0.0005 (14)	0.0006 (13)	0.0013 (13)
C22	0.0244 (18)	0.0243 (18)	0.0217 (16)	0.0018 (14)	0.0015 (13)	−0.0005 (13)
C23	0.0263 (19)	0.036 (2)	0.0326 (19)	−0.0013 (16)	0.0095 (15)	0.0031 (16)
C24	0.033 (2)	0.042 (2)	0.0265 (18)	−0.0049 (17)	0.0088 (15)	0.0060 (16)
C25	0.032 (2)	0.033 (2)	0.0243 (18)	−0.0030 (16)	0.0014 (15)	0.0070 (15)
C26	0.038 (2)	0.058 (3)	0.042 (2)	0.003 (2)	0.0017 (19)	0.026 (2)
C27	0.0258 (19)	0.032 (2)	0.0281 (18)	0.0048 (15)	−0.0012 (15)	0.0067 (15)
C28	0.0259 (19)	0.041 (2)	0.0300 (19)	0.0034 (17)	0.0055 (15)	−0.0032 (16)
C29	0.080 (4)	0.075 (4)	0.035 (2)	0.016 (3)	−0.006 (2)	0.007 (2)
C30	0.045 (3)	0.107 (5)	0.053 (3)	−0.023 (3)	0.013 (2)	−0.039 (3)
N1	0.0232 (15)	0.0209 (14)	0.0210 (13)	0.0001 (11)	0.0022 (11)	0.0026 (11)
N2	0.0216 (14)	0.0211 (14)	0.0197 (13)	−0.0001 (11)	0.0034 (11)	0.0011 (11)
N3	0.0267 (16)	0.0348 (17)	0.0238 (15)	−0.0005 (13)	−0.0006 (12)	−0.0027 (13)
N4	0.0211 (15)	0.0235 (15)	0.0236 (14)	0.0012 (12)	0.0010 (11)	0.0041 (11)
N5	0.0220 (15)	0.0279 (15)	0.0197 (13)	0.0001 (12)	0.0015 (11)	0.0029 (12)
N6	0.0271 (17)	0.055 (2)	0.0313 (17)	0.0066 (15)	0.0014 (13)	−0.0081 (16)
O1	0.0349 (15)	0.0336 (14)	0.0220 (12)	−0.0056 (11)	0.0037 (10)	−0.0001 (11)
O2	0.0383 (16)	0.0420 (16)	0.0289 (13)	−0.0062 (13)	0.0002 (12)	−0.0016 (12)
O3	0.066 (3)	0.056 (2)	0.153 (4)	0.007 (2)	0.023 (3)	0.036 (3)
Cl1	0.0392 (5)	0.0567 (6)	0.0198 (4)	−0.0049 (5)	0.0021 (4)	−0.0024 (4)
Cl2	0.0199 (4)	0.0461 (6)	0.0344 (5)	0.0039 (4)	0.0040 (3)	0.0086 (4)
Cl3	0.0316 (5)	0.0272 (5)	0.0364 (5)	0.0020 (4)	0.0097 (4)	0.0079 (4)
Cl4	0.0338 (5)	0.0286 (5)	0.0276 (4)	−0.0034 (4)	0.0071 (4)	0.0028 (3)
Cl5	0.0327 (5)	0.0588 (7)	0.0450 (5)	0.0229 (5)	0.0101 (4)	0.0232 (5)
Cl6	0.0294 (5)	0.0353 (5)	0.0409 (5)	−0.0057 (4)	0.0085 (4)	0.0084 (4)
In1	0.01898 (13)	0.02579 (14)	0.01822 (11)	0.00006 (10)	0.00357 (9)	0.00353 (9)
In2	0.01887 (13)	0.02634 (14)	0.02381 (13)	0.00307 (10)	0.00239 (9)	0.00571 (10)

### Geometric parameters (Å, º)

**Table:**

C1—N1	1.340 (4)	C18—H18C	0.9600
C1—C2	1.393 (5)	C19—C20	1.378 (5)
C1—H1	0.9300	C19—H19	0.9300
C2—C4	1.383 (5)	C20—C21	1.381 (5)
C2—C3	1.504 (5)	C20—H20	0.9300
C3—H3C	0.9600	C21—N4	1.349 (4)
C3—H3B	0.9600	C21—C22	1.490 (5)
C3—H3A	0.9600	C22—N5	1.337 (4)
C4—C5	1.386 (5)	C22—C23	1.385 (5)
C4—H4	0.9300	C23—C24	1.389 (6)
C5—C6	1.396 (5)	C23—H23	0.9300
C5—H5	0.9300	C24—C25	1.380 (6)
C6—N1	1.342 (4)	C24—H24	0.9300
C6—C7	1.487 (5)	C25—C27	1.387 (5)
C7—N2	1.352 (4)	C25—C26	1.501 (5)
C7—C8	1.388 (5)	C26—H26C	0.9600
C8—C9	1.381 (5)	C26—H26B	0.9600
C8—H8	0.9300	C26—H26A	0.9600
C9—C10	1.395 (5)	C27—N5	1.341 (4)
C9—H9	0.9300	C27—H27	0.9300
C10—C12	1.384 (5)	C28—O2	1.262 (4)
C10—C11	1.496 (5)	C28—N6	1.293 (5)
C11—H11C	0.9600	C28—H28	0.9300
C11—H11B	0.9600	C29—N6	1.452 (6)
C11—H11A	0.9600	C29—H29C	0.9600
C12—N2	1.343 (4)	C29—H29B	0.9600
C12—H12	0.9300	C29—H29A	0.9600
C13—O1	1.248 (4)	C30—N6	1.456 (6)
C13—N3	1.309 (4)	C30—H30A	0.9600
C13—H13	0.9300	C30—H30B	0.9600
C14—N3	1.469 (5)	C30—H30C	0.9600
C14—H14C	0.9600	O3—H3D	0.844 (10)
C14—H14B	0.9600	O3—H3E	0.841 (10)
C14—H14A	0.9600	In1—Cl1	2.4185 (9)
C15—N3	1.454 (5)	In1—Cl2	2.4227 (9)
C15—H15A	0.9600	In1—Cl3	2.4496 (9)
C15—H15B	0.9600	In1—O1	2.267 (2)
C15—H15C	0.9600	In1—N1	2.287 (3)
C16—N4	1.333 (4)	In1—N2	2.301 (3)
C16—C17	1.387 (5)	In2—Cl4	2.4737 (9)
C16—H16	0.9300	In2—Cl5	2.4327 (10)
C17—C19	1.388 (5)	In2—Cl6	2.4326 (9)
C17—C18	1.502 (5)	In2—O2	2.202 (3)
C18—H18A	0.9600	In2—N4	2.292 (3)
C18—H18B	0.9600	In2—N5	2.316 (3)
			
N1—C1—C2	123.4 (3)	C24—C23—H23	120.4
N1—C1—H1	118.3	C25—C24—C23	120.2 (3)
C2—C1—H1	118.3	C25—C24—H24	119.9
C4—C2—C1	116.6 (3)	C23—C24—H24	119.9
C4—C2—C3	121.7 (3)	C24—C25—C27	116.7 (3)
C1—C2—C3	121.6 (3)	C24—C25—C26	122.0 (3)
C2—C3—H3C	109.5	C27—C25—C26	121.3 (4)
C2—C3—H3B	109.5	C25—C26—H26C	109.5
H3C—C3—H3B	109.5	C25—C26—H26B	109.5
C2—C3—H3A	109.5	H26C—C26—H26B	109.5
H3C—C3—H3A	109.5	C25—C26—H26A	109.5
H3B—C3—H3A	109.5	H26C—C26—H26A	109.5
C2—C4—C5	120.8 (3)	H26B—C26—H26A	109.5
C2—C4—H4	119.6	N5—C27—C25	123.7 (3)
C5—C4—H4	119.6	N5—C27—H27	118.2
C4—C5—C6	118.8 (3)	C25—C27—H27	118.2
C4—C5—H5	120.6	O2—C28—N6	122.5 (4)
C6—C5—H5	120.6	O2—C28—H28	118.7
N1—C6—C5	120.9 (3)	N6—C28—H28	118.7
N1—C6—C7	116.8 (3)	N6—C29—H29C	109.5
C5—C6—C7	122.2 (3)	N6—C29—H29B	109.5
N2—C7—C8	120.9 (3)	H29C—C29—H29B	109.5
N2—C7—C6	116.7 (3)	N6—C29—H29A	109.5
C8—C7—C6	122.4 (3)	H29C—C29—H29A	109.5
C9—C8—C7	119.5 (3)	H29B—C29—H29A	109.5
C9—C8—H8	120.3	N6—C30—H30A	109.5
C7—C8—H8	120.3	N6—C30—H30B	109.5
C8—C9—C10	120.1 (3)	H30A—C30—H30B	109.5
C8—C9—H9	119.9	N6—C30—H30C	109.5
C10—C9—H9	119.9	H30A—C30—H30C	109.5
C12—C10—C9	116.8 (3)	H30B—C30—H30C	109.5
C12—C10—C11	122.6 (3)	C1—N1—C6	119.4 (3)
C9—C10—C11	120.6 (3)	C1—N1—In1	123.7 (2)
C10—C11—H11C	109.5	C6—N1—In1	116.7 (2)
C10—C11—H11B	109.5	C12—N2—C7	118.8 (3)
H11C—C11—H11B	109.5	C12—N2—In1	124.1 (2)
C10—C11—H11A	109.5	C7—N2—In1	115.0 (2)
H11C—C11—H11A	109.5	C13—N3—C15	121.6 (3)
H11B—C11—H11A	109.5	C13—N3—C14	121.2 (3)
N2—C12—C10	123.8 (3)	C15—N3—C14	117.2 (3)
N2—C12—H12	118.1	C16—N4—C21	119.0 (3)
C10—C12—H12	118.1	C16—N4—In2	123.1 (2)
O1—C13—N3	122.3 (3)	C21—N4—In2	117.8 (2)
O1—C13—H13	118.8	C22—N5—C27	119.1 (3)
N3—C13—H13	118.8	C22—N5—In2	117.4 (2)
N3—C14—H14C	109.5	C27—N5—In2	123.2 (2)
N3—C14—H14B	109.5	C28—N6—C29	120.8 (4)
H14C—C14—H14B	109.5	C28—N6—C30	121.4 (4)
N3—C14—H14A	109.5	C29—N6—C30	117.8 (4)
H14C—C14—H14A	109.5	C13—O1—In1	126.5 (2)
H14B—C14—H14A	109.5	C28—O2—In2	132.3 (3)
N3—C15—H15A	109.5	H3D—O3—H3E	103.2 (16)
N3—C15—H15B	109.5	O1—In1—N1	80.31 (9)
H15A—C15—H15B	109.5	O1—In1—N2	74.40 (9)
N3—C15—H15C	109.5	N1—In1—N2	72.49 (10)
H15A—C15—H15C	109.5	O1—In1—Cl1	168.73 (7)
H15B—C15—H15C	109.5	N1—In1—Cl1	89.31 (7)
N4—C16—C17	124.0 (3)	N2—In1—Cl1	98.40 (7)
N4—C16—H16	118.0	O1—In1—Cl2	90.23 (7)
C17—C16—H16	118.0	N1—In1—Cl2	165.72 (7)
C16—C17—C19	116.1 (3)	N2—In1—Cl2	94.75 (7)
C16—C17—C18	122.3 (3)	Cl1—In1—Cl2	99.11 (3)
C19—C17—C18	121.5 (3)	O1—In1—Cl3	87.14 (7)
C17—C18—H18A	109.5	N1—In1—Cl3	92.18 (7)
C17—C18—H18B	109.5	N2—In1—Cl3	157.55 (7)
H18A—C18—H18B	109.5	Cl1—In1—Cl3	97.68 (4)
C17—C18—H18C	109.5	Cl2—In1—Cl3	98.06 (3)
H18A—C18—H18C	109.5	O2—In2—N4	88.49 (10)
H18B—C18—H18C	109.5	O2—In2—N5	76.22 (10)
C20—C19—C17	120.7 (3)	N4—In2—N5	71.49 (10)
C20—C19—H19	119.6	O2—In2—Cl6	89.75 (7)
C17—C19—H19	119.6	N4—In2—Cl6	92.52 (7)
C19—C20—C21	119.3 (4)	N5—In2—Cl6	158.71 (8)
C19—C20—H20	120.4	O2—In2—Cl5	89.31 (8)
C21—C20—H20	120.4	N4—In2—Cl5	162.54 (7)
N4—C21—C20	120.9 (3)	N5—In2—Cl5	91.17 (8)
N4—C21—C22	116.6 (3)	Cl6—In2—Cl5	104.79 (4)
C20—C21—C22	122.6 (3)	O2—In2—Cl4	173.50 (8)
N5—C22—C23	121.1 (3)	N4—In2—Cl4	87.98 (7)
N5—C22—C21	116.6 (3)	N5—In2—Cl4	97.47 (7)
C23—C22—C21	122.3 (3)	Cl6—In2—Cl4	95.86 (3)
C22—C23—C24	119.2 (4)	Cl5—In2—Cl4	92.42 (4)
C22—C23—H23	120.4		
			
N1—C1—C2—C4	−1.2 (5)	C23—C22—N5—In2	174.8 (3)
N1—C1—C2—C3	179.1 (3)	C21—C22—N5—In2	−4.7 (4)
C1—C2—C4—C5	1.4 (5)	C25—C27—N5—C22	−2.1 (5)
C3—C2—C4—C5	−178.9 (3)	C25—C27—N5—In2	−175.4 (3)
C2—C4—C5—C6	−0.8 (5)	O2—C28—N6—C29	−4.9 (6)
C4—C5—C6—N1	−0.1 (5)	O2—C28—N6—C30	174.8 (4)
C4—C5—C6—C7	−177.1 (3)	N3—C13—O1—In1	−172.6 (3)
N1—C6—C7—N2	−12.9 (4)	N6—C28—O2—In2	151.2 (3)
C5—C6—C7—N2	164.3 (3)	C13—O1—In1—N1	−130.2 (3)
N1—C6—C7—C8	166.3 (3)	C13—O1—In1—N2	155.4 (3)
C5—C6—C7—C8	−16.5 (5)	C13—O1—In1—Cl1	−153.3 (3)
N2—C7—C8—C9	−0.5 (5)	C13—O1—In1—Cl2	60.6 (3)
C6—C7—C8—C9	−179.7 (3)	C13—O1—In1—Cl3	−37.5 (3)
C7—C8—C9—C10	−1.8 (5)	C1—N1—In1—O1	113.0 (3)
C8—C9—C10—C12	2.0 (5)	C6—N1—In1—O1	−71.1 (2)
C8—C9—C10—C11	−178.8 (3)	C1—N1—In1—N2	−170.4 (3)
C9—C10—C12—N2	0.2 (5)	C6—N1—In1—N2	5.5 (2)
C11—C10—C12—N2	−179.1 (3)	C1—N1—In1—Cl1	−71.4 (2)
N4—C16—C17—C19	1.2 (5)	C6—N1—In1—Cl1	104.5 (2)
N4—C16—C17—C18	−177.6 (3)	C1—N1—In1—Cl2	162.2 (2)
C16—C17—C19—C20	−0.7 (5)	C6—N1—In1—Cl2	−22.0 (5)
C18—C17—C19—C20	178.2 (3)	C1—N1—In1—Cl3	26.3 (2)
C17—C19—C20—C21	0.1 (6)	C6—N1—In1—Cl3	−157.8 (2)
C19—C20—C21—N4	0.1 (5)	C12—N2—In1—O1	−91.2 (3)
C19—C20—C21—C22	179.1 (3)	C7—N2—In1—O1	72.3 (2)
N4—C21—C22—N5	3.7 (4)	C12—N2—In1—N1	−175.7 (3)
C20—C21—C22—N5	−175.3 (3)	C7—N2—In1—N1	−12.3 (2)
N4—C21—C22—C23	−175.8 (3)	C12—N2—In1—Cl1	97.7 (2)
C20—C21—C22—C23	5.2 (5)	C7—N2—In1—Cl1	−98.9 (2)
N5—C22—C23—C24	−0.2 (5)	C12—N2—In1—Cl2	−2.2 (2)
C21—C22—C23—C24	179.3 (3)	C7—N2—In1—Cl2	161.2 (2)
C22—C23—C24—C25	0.1 (6)	C12—N2—In1—Cl3	−126.9 (2)
C23—C24—C25—C27	−1.0 (5)	C7—N2—In1—Cl3	36.5 (3)
C23—C24—C25—C26	−179.9 (4)	C28—O2—In2—N4	111.2 (4)
C24—C25—C27—N5	2.1 (5)	C28—O2—In2—N5	−177.5 (4)
C26—C25—C27—N5	−179.1 (4)	C28—O2—In2—Cl6	18.7 (4)
C2—C1—N1—C6	0.4 (5)	C28—O2—In2—Cl5	−86.1 (4)
C2—C1—N1—In1	176.1 (2)	C28—O2—In2—Cl4	168.4 (5)
C5—C6—N1—C1	0.3 (5)	C16—N4—In2—O2	−107.4 (3)
C7—C6—N1—C1	177.5 (3)	C21—N4—In2—O2	74.9 (2)
C5—C6—N1—In1	−175.7 (2)	C16—N4—In2—N5	176.6 (3)
C7—C6—N1—In1	1.4 (3)	C21—N4—In2—N5	−1.0 (2)
C10—C12—N2—C7	−2.5 (5)	C16—N4—In2—Cl6	−17.7 (3)
C10—C12—N2—In1	160.4 (3)	C21—N4—In2—Cl6	164.6 (2)
C8—C7—N2—C12	2.6 (5)	C16—N4—In2—Cl5	169.7 (2)
C6—C7—N2—C12	−178.2 (3)	C21—N4—In2—Cl5	−8.0 (4)
C8—C7—N2—In1	−161.8 (2)	C16—N4—In2—Cl4	78.1 (3)
C6—C7—N2—In1	17.5 (3)	C21—N4—In2—Cl4	−99.6 (2)
O1—C13—N3—C15	2.1 (6)	C22—N5—In2—O2	−89.9 (2)
O1—C13—N3—C14	−175.8 (4)	C27—N5—In2—O2	83.5 (3)
C17—C16—N4—C21	−1.1 (5)	C22—N5—In2—N4	3.1 (2)
C17—C16—N4—In2	−178.7 (3)	C27—N5—In2—N4	176.5 (3)
C20—C21—N4—C16	0.4 (5)	C22—N5—In2—Cl6	−39.9 (4)
C22—C21—N4—C16	−178.7 (3)	C27—N5—In2—Cl6	133.5 (2)
C20—C21—N4—In2	178.1 (3)	C22—N5—In2—Cl5	−178.9 (2)
C22—C21—N4—In2	−0.9 (4)	C27—N5—In2—Cl5	−5.5 (3)
C23—C22—N5—C27	1.1 (5)	C22—N5—In2—Cl4	88.5 (2)
C21—C22—N5—C27	−178.4 (3)	C27—N5—In2—Cl4	−98.1 (3)

### Hydrogen-bond geometry (Å, º)

**Table:**

*D*—H···*A*	*D*—H	H···*A*	*D*···*A*	*D*—H···*A*
O3—H3*D*···Cl5^i^	0.84 (3)	2.49 (3)	3.326 (5)	174 (3)
O3—H3*E*···Cl3^ii^	0.84 (2)	2.40 (2)	3.183 (5)	156 (3)
C5—H5···Cl4	0.93	2.69	3.620 (3)	175
C8—H8···Cl4	0.93	2.69	3.574 (4)	160
C9—H9···Cl4^iii^	0.93	2.78	3.657 (3)	158
C16—H16···Cl6	0.93	2.80	3.430 (4)	126
C20—H20···O3^iv^	0.93	2.60	3.526 (6)	178
C23—H23···O3^iv^	0.93	2.49	3.416 (6)	173
C27—H27···Cl5	0.93	2.71	3.370 (4)	129

Symmetry codes: (i) −*x*, −*y*+1, −*z*; (ii) −*x*+1, −*y*+2, −*z*; (iii) −*x*+1, −*y*+1, −*z*+1; (iv) −*x*+1, −*y*+1, −*z*.

## References

[bb1] Albada, G. A., Mohamadou, A., Mutikainen, I., Turpeinen, U. & Reedijk, J. (2004). *Eur. J. Inorg. Chem.* pp. 3733–3742.

[bb2] Alizadeh, R., Amani, V., Farshady, A. A. & Khavasi, H. R. (2010). *J. Coord. Chem.* **63**, 2122–2131.

[bb3] Amani, V., Safari, N. & Khavasi, H. R. (2007). *Polyhedron*, **26**, 4257–4262.

[bb4] Amani, V., Safari, N., Khavasi, H. R. & Akkurt, M. (2009). *Polyhedron*, **28**, 3026–3030.

[bb5] Bruker (2001). *SADABS* Bruker AXS Inc., Madison, Wisconsin, USA.

[bb6] Bruker (2007). *APEX2* and *SAINT* Bruker AXS Inc., Madison, Wisconsin, USA.

[bb7] Kalateh, K., Ahmadi, R., Ebadi, A., Amani, V. & Khavasi, H. R. (2008). *Acta Cryst.* E**64**, m1353–m1354. 10.1107/S160053680803119X PMC2959790 21580816

[bb8] Khalighi, A., Ahmadi, R., Amani, V. & Khavasi, H. R. (2008). *Acta Cryst.* E**64**, m1211–m1212. 10.1107/S1600536808027104 PMC2960607 21201646

[bb9] Sheldrick, G. M. (2008). *Acta Cryst.* A**64**, 112–122. 10.1107/S0108767307043930 18156677

[bb10] Shirvan, S. A. & Haydari Dezfuli, S. (2012). *Acta Cryst.* E**68**, m846. 10.1107/S1600536812023860 PMC3379185 22719383

[bb11] Tadayon Pour, N., Ebadi, A., Abedi, A., Amani, V. & Khavasi, H. R. (2008). *Acta Cryst.* E**64**, m1305. 10.1107/S160053680802953X PMC2959332 21201044

